# Brittle cornea syndrome: recognition, molecular diagnosis and management

**DOI:** 10.1186/1750-1172-8-68

**Published:** 2013-05-04

**Authors:** Emma MM Burkitt Wright, Louise F Porter, Helen L Spencer, Jill Clayton-Smith, Leon Au, Francis L Munier, Sarah Smithson, Mohnish Suri, Marianne Rohrbach, Forbes DC Manson, Graeme CM Black

**Affiliations:** 1 Genetic Medicine, Institute of Human Development, Faculty of Medical and Human Sciences, University of Manchester, Manchester, UK; 2 Genetic Medicine, St Mary’s Hospital, Central Manchester University Hospitals NHS Foundation Trust, Manchester Academic Health Science Centre, Manchester, UK; 3 University of Bristol Faculty of Medicine and Dentistry, Bristol Royal Infirmary, Bristol, UK; 4 Royal Manchester Eye Hospital, Central Manchester University Hospitals NHS Foundation Trust, Oxford Road, Manchester, M13 9WL, UK; 5 Ophthalmology, Jules-Gonin Eye Hospital, Lausanne, Switzerland; 6 Clinical Genetics, University Hospitals Bristol NHS Foundation Trust, Bristol, UK; 7 Clinical Genetics Service, Nottingham University Hospitals NHS Trust, City Hospital Campus, Hucknall Road, Nottingham, NG5 1PB, UK; 8 Division of Metabolism, Connective Tissue Unit, University Children's Hospital, Steinwiessstrasse 75, Zurich, 8032, Switzerland

**Keywords:** Brittle cornea syndrome, Corneal rupture, Blue sclera, ZNF469, PRDM5

## Abstract

Brittle cornea syndrome (BCS) is an autosomal recessive disorder characterised by extreme corneal thinning and fragility. Corneal rupture can therefore occur either spontaneously or following minimal trauma in affected patients. Two genes, *ZNF469* and *PRDM5*, have now been identified, in which causative pathogenic mutations collectively account for the condition in nearly all patients with BCS ascertained to date. Therefore, effective molecular diagnosis is now available for affected patients, and those at risk of being heterozygous carriers for BCS. We have previously identified mutations in *ZNF469* in 14 families (in addition to 6 reported by others in the literature), and in *PRDM5* in 8 families (with 1 further family now published by others). Clinical features include extreme corneal thinning with rupture, high myopia, blue sclerae, deafness of mixed aetiology with hypercompliant tympanic membranes, and variable skeletal manifestations. Corneal rupture may be the presenting feature of BCS, and it is possible that this may be incorrectly attributed to non-accidental injury. Mainstays of management include the prevention of ocular rupture by provision of protective polycarbonate spectacles, careful monitoring of visual and auditory function, and assessment for skeletal complications such as developmental dysplasia of the hip. Effective management depends upon appropriate identification of affected individuals, which may be challenging given the phenotypic overlap of BCS with other connective tissue disorders.

## Background

Brittle cornea syndrome (BCS) is an autosomal recessive condition that results from pathogenic variants in one of two genes, *ZNF469*[1,2] and *PRDM5*[3,4]. BCS is a rare condition, although the recent identification of mutations in patients with thin corneas as part of a generalised connective tissue disorder not previously clinically defined as BCS [3] suggest that it may be under-diagnosed. The recent identification of a patient with BCS with an ocular-only phenotype [5] also supports this hypothesis, as does the extremely high proportion of patients identified to date who have consanguineous parentage. Whilst the very high risk of ocular rupture is the most characteristic feature of BCS, it is a multisystem connective tissue disorder, and a variety of extra-ocular manifestations have been reported, particularly deafness [3]. Due to only recent identification of the causative genes for BCS, no previous article has addressed the clinical presentation and management of this condition in patients with a molecular diagnosis, or been able to assess for genotype-phenotype correlation. Here we summarise phenotypic data from affected patients, and make recommendations for clinical management (including molecular diagnosis) based on past experience and currently available evidence.

## Methods

Patients whose data we included in this study were those in whom mutations in *PRDM5* and *ZNF469* have been identified in our laboratories as previously described [3,6], and others published in the scientific literature whose mutational status has been published. Clinical and molecular data were assessed for evidence of genotype-phenotype correlations. Approval was granted by North Manchester NHS Research Ethics Committee, reference 06/1406/52, and written consent was obtained from participants or their parents/guardians.

## Results

### Ophthalmic phenotypes

The striking ocular phenotype of BCS serves to distinguish it from other similar conditions including many forms of Ehlers-Danlos Syndrome (EDS). Clinical features are listed in the Summary of common features in patients with BCS. In its classical form, BCS is characterised by extreme corneal fragility and thinning, often leading to corneal perforation either spontaneously or after minor trauma [1,7,8]. Corneal perforation is frequently observed as young as 2–3 years of age in BCS, emphasising the critical importance of early identification of this condition. As a generalised connective tissue disorder, involvement of multiple tissues can usually be demonstrated, but the recent identification of a single patient with BCS due to a homozygous frame shift mutation in *ZNF469* and no extra-ocular manifestations by the age of 37 years [5] is notable as it suggests that such mutations could cause non-syndromic presentations in other patients, and therefore, potentially be a rare cause of keratoconus or apparently isolated corneal rupture.

Summary of common features in patients with BCS

OPHTHALMIC

Thin cornea, with or without rupture (central corneal thickness often <400 μm)

Enucleation or corneal scarring as a result of previous rupture

Progressive loss of corneal stromal depth, especially in central cornea

Blue sclera

Early onset progressive keratoconus and/or keratoglobus

High myopia, with normal or moderately increased axial length

Retinal detachment

AUDITORY

Deafness, often with mixed conductive and sensorineural components

Hypercompliant tympanic membranes

Progressive deafness, higher frequencies often more severely affected (‘sloping’ pure tone audiogram)

MUSCULOSKELETAL

Developmental dysplasia of the hip

Hypotonia in infancy, usually mild if present

Scoliosis

Arachnodactyly

Small joint hypermobility, pes planus, hallux valgus

Mild contractures of fingers (especially 5^th^)

SKIN

Soft, doughy skin; other skin manifestations (hyperelasticity, abnormal scarring) are usually mild if present

**Figure 1:**
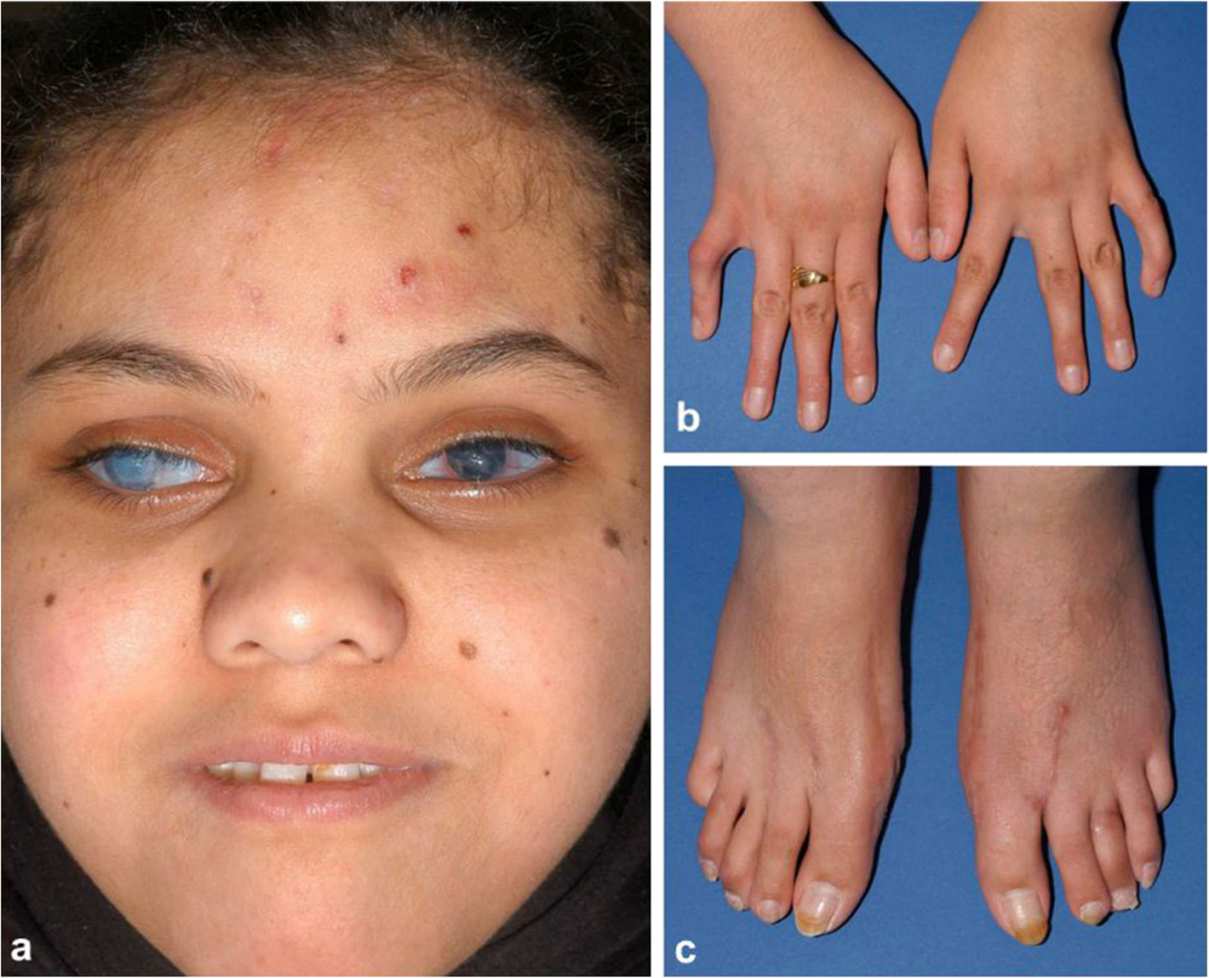
**Clinical appearance of patient with BCS.** This patient has a homozygous *ZNF469* c.6444delG mutation (patient P3, Rohrbach et al. [6]) and is pictured at 18 years of age: **a**) face, **b**) hands and **c**) feet. Note extensive corneal scarring bilaterally and blue sclerae. Her facial appearance is reminiscent of several other patients with BCS, with a short nose, but otherwise unremarkable morphology. Several naevi are present over her cheeks and the lesions on her forehead are small scabs. In the hands, bilateral clinodactyly is seen, and in the feet, pes planus and scars of previous surgical management of hallux valgus.

Nearly all patients with BCS become blind from complications of corneal perforation and resultant scarring (Figure 1a). Other ocular features that affect vision are keratoconus, keratoglobus and high myopia. These occur because BCS corneas are unable to withstand normal biomechanical stresses and hence cannot maintain their structural integrity and shape. A high index of suspicion may be required to make the diagnosis in milder cases in which corneal thinning has not yet led to rupture [1,7,9].

**Figure 2:**
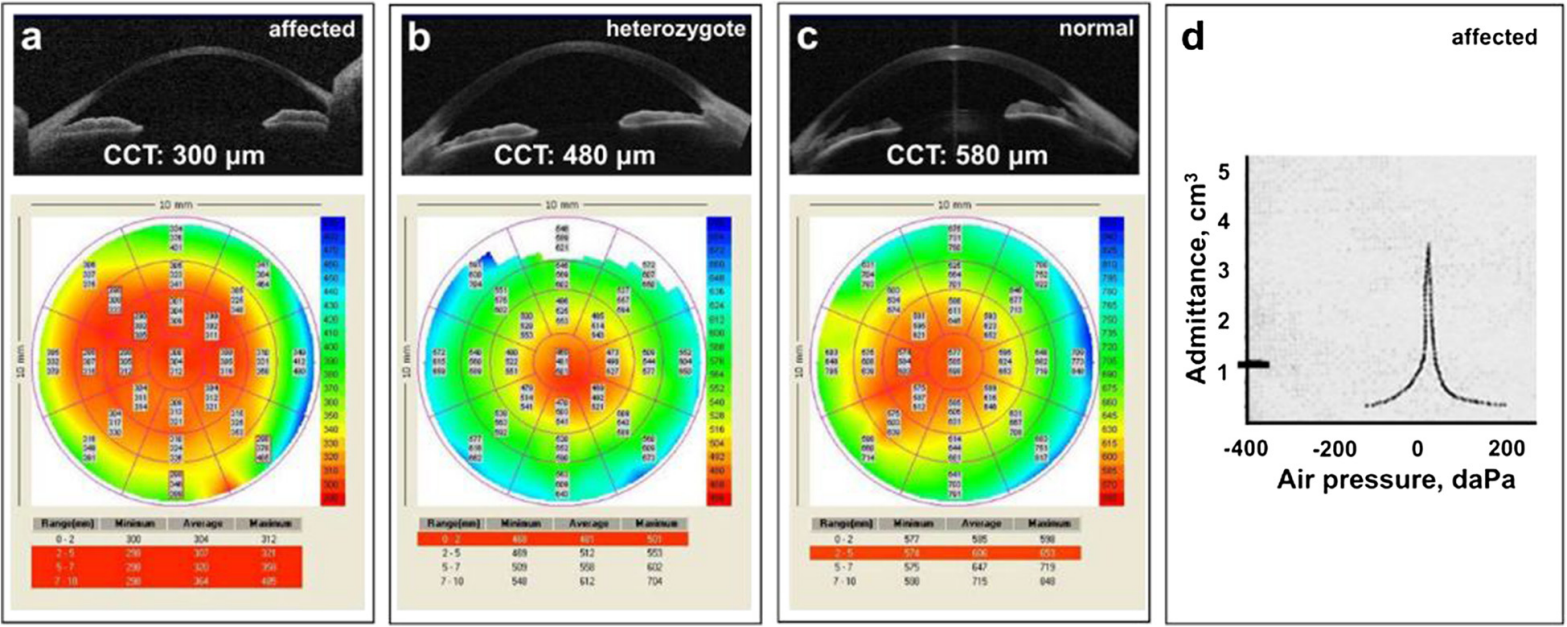
**Investigations in BCS.** (**a**) Ocular optical coherence tomography (OCT) and pachymetry of a 32 year old patient with homozygous mutation in *PRDM5* (deletion of exons 11–16; IV:4 of family BCS-001, Burkitt Wright et al. [3]). Note extreme thinning, particularly of the central cornea (300 μm) but even the maximum thickness at the periphery is only 330–380 μm. (**b**) Ocular OCT and pachymetry of a 31 year old patient with heterozygous mutation in *PRDM5* (IV:8, BCS-001). Note only mild corneal thinning on OCT, with mean CCT measurement of 480 μm, increasing to 580–620 μm at the periphery. (**c**) Ocular OCT and pachymetry of a normal eye for comparison with a) and b), showing CCT of 580 μm and peripheral thickness of 650–750 μm. (**d**) Tympanogram of BCS patient (IV:4, BCS-001): a Type Ad curve is observed, demonstrating normal middle ear pressure but hypercompliance of the tympanic membrane. The volume by which the eardrum is displaced when a pulse of pressure is delivered to it is represented by the curve (~3.5 cm^3^), whilst the marker on the left demonstrates the degree of compliance observed (~1 cm^3^) for a normal tympanic membrane.

Where it has been possible to fully assess corneal thinning, the large majority of patients with BCS have had central corneal thickness (CCT) of less than 400 μm (normal range 515–575 μm [10]). On pachymetry, thinning has been most pronounced in the central cornea, with relative preservation of peripheral corneal thickness (Figure 2a).

Blue sclerae are usually present in those affected by BCS, but this is not universal [5], may disappear over time, [11] is also present in many individuals identified to be heterozygous carriers for BCS-associated mutations [3,7], and may also occur in normal individuals. The presence of a blue sclera is believed to correlate with a reduction in scleral thickness of at least one third [8].

Further ophthalmic features have been reported in BCS. Secondary glaucoma has been reported in many affected patients, particularly those with extensive corneal damage following rupture. One such patient, who was in his fifth decade, whose eyes had axial lengths of 26.7 mm and 22.5 mm (normal range: 21.5 – 23 mm) had also suffered bilateral retinal detachments (patient IV:6 in Christensen et al. [2]). Retinal detachments were also noted in one other patient with BCS, a 5-year old boy diagnosed clinically in the pre-molecular era [12]. Retinal detachment does not appear to be a common ocular feature of BCS, but this observation is influenced by the early age at which most patients have been examined or had to undergo enucleation. Secondary glaucoma as a contributor to blindness had not been previously highlighted in BCS, but is now increasingly recognised [6].

Individuals heterozygous for BCS-associated mutations have been reported to have a relevant phenotype, including blue sclerae and small joint hypermobility [3]. These are not always present, and in particular may not be striking in adult carriers, but nonetheless can cause diagnostic confusion, particularly in multiply consanguineous families where there may be potentially at-risk individuals across several generations. Heterozygous carriers for BCS, where formally assessed, have often been found to have mild corneal thinning, for example CCT measurements around 500 μm (Figure 2b), which may prove a further limitation to accurate clinical confirmation of disease status. Keratoconus has also been diagnosed in a young adult known to be heterozygous for a mutation in *PRDM5* (IV:8, BCS-001, of Burkitt Wright et al. [3]).

### Auditory phenotype

The audiological features of brittle cornea syndrome are less frequently reported and of less dramatic onset than many of the ophthalmic complications, and hence have not yet been as comprehensively studied. The largest previous survey of affected patients, collated in the pre-molecular era [13] suggested that at least one third may have significant problems with hearing, including severe to profound deafness in some individuals. This is borne out by the identification of variable degrees of deafness in three of seven families with *PRDM5*-associated BCS, including all affected individuals in families BCS-001 and BCS-002 in which mutations were first identified [3]. The degrees of deafness in these two families ranged from a hearing loss of 25–30 dB mild enough to require no intervention in a 30 year old woman (IV:6 of BCS-002), to severe bilateral deafness in early childhood (IV:9 of BCS-001). Deafness was also present in two of three unrelated comprehensively assessed patients with *ZNF469* mutations [6].

Both inter- and intra-familial variability with respect to age of onset and progression of deafness have been observed, and a mixed aetiology with sensorineural and conductive components has often been suggested, with conductive components predominating in childhood. In keeping with this, some patients have derived benefit from tympanostomy in childhood, but others have not had evidence of glue ear, and hypermobility of the ossicular chain has been implicated [3].

Where tympanometry has been performed, hypercompliant tympanic membranes, as may be seen also in association with other generalised connective tissue disorders, have been demonstrated in a very high proportion of patients (Figure 2d). Hypercompliant tympanic membranes are thought to be caused by a combination of factors, including increased intrinsic compliance of the eardrum itself, and joint laxity between the ossicles of the middle ear [14]. Tympanometry is a non-invasive test routinely available in audiological medicine and may therefore be a useful diagnostic procedure to assess the likelihood of BCS. The identification of hypercompliant tympanic membranes should prompt further consideration of BCS as a possible diagnosis. No evidence of hearing loss or abnormal tympanometry in heterozygous individuals has yet been identified.

### Other features

In keeping with a generalised connective tissue disorder, musculoskeletal features have been present in many patients with BCS, most notably developmental dysplasia of the hip (DDH) and scoliosis. DDH or other hip abnormalities in childhood have been present in several affected individuals, for example all four affected siblings of BCS-001 and two of four affected individuals of BCS-002 [3]. Scoliosis also affects a significant proportion of patients, such as those of Christensen et al. [2]. Reduced bone mineral density has been observed in adults with BCS due to *PRDM5*[3] or *ZNF469* mutations [2], even amongst those with normal Vitamin D status. Repeated fractures have also been reported in a significant minority of affected patients.

Small joint hypermobility has been notable in the majority of affected individuals and present to a milder degree in many of their relatives without BCS (especially those who were heterozygous carriers for the causative mutation). Mild contractures of the 5^th^ fingers have been noted in several patients (Figure 1b), but have not usually required active treatment in this young patient cohort. Hallux valgus (Figure 1c), a common finding in the general population, has again been noted in several people with BCS, including with an unusually early age of onset. The patient in Figure 1, P3 in Rohrbach et al’s series, [6] required surgery aged 14 years to manage severe deformity. Easy bruising has been noted in many affected individuals, but skin healing and scarring have usually appeared to be normal or only mildly altered.

Obstetric and perinatal problems reminiscent of those seen in other connective tissue disorders have also been present in several individuals with BCS: premature birth after premature rupture of membranes (V: 1, BCS-002), and primary primiparous cervical incompetence, resulting in second trimester pregnancy loss (IV:4, BCS-001) have each been observed in individuals with mutations in *PRDM5*[3].

Mitral valve dysfunction and cardiovascular symptomatology has also been identified in more than one individual with BCS, and echocardiography may therefore be warranted in this patient group. Importantly, vascular ruptures as have occurred in EDS VI (Kyphoscoliotic type) [15] have not been reported in BCS patients to date.

## Discussion

### Differential diagnosis

Differential diagnoses of BCS are summarised in Table 1. These have long been known to include Ehlers-Danlos syndrome (EDS) type VI (OMIM: 225400), formerly described as EDS VIA. Indeed, BCS has previously, on occasion, been termed EDS VIB, however, this nomenclature has also been used for a range of other phenotypes that, like BCS, show a normal LP:HP ratio, but are genetically and, usually, clinically, distinct from it, such as the musculocontractural form of EDS (OMIM: 601776). This situation suggests that BCS remains best classified as a separate entity.

**Table 1:** Differential diagnosis of BCS: autosomal recessive connective tissue disorders with blue sclera and thin cornea

**Condition / phenotype**	**OMIM**	**Gene**	**Protein**	**OMIM**
BCS	229200	*ZNF469*	Zinc finger protein 469	612078
	614170	*PRDM5*	PR domain containing 5	614161
EDS VI	225400	*PLOD1*	Lysyl hydroxylase 1	153454
EDS, musculocontractural type	601776	*CHST14*	Carbohydrate sulfotransferase 14	608429
EDS with progressive kyphoscoliosis, myopathy and hearing loss	614557	*FKBP14*	FK506 binding protein 14	614505
Bone fragility with contractures, arterial rupture and deafness	612394	*PLOD3*	Lysyl hydroxylase 3	603066
Spondylocheiro dysplastic type of EDS	612350	*SLC39A13*	ZIP3	608735

Other, rare, autosomal recessive forms of Ehlers Danlos syndrome (EDS) and osteogenesis imperfecta (OI) have also been characterised, but these would be unlikely to present within the differential diagnosis of BCS, for example the dermatosparactic form of EDS (VIIc, OMIM 604539, due to mutations in *ADAMTS2*[39]) has dramatic skin manifestations not seen to date in BCS patients, whilst the extremely rare patients with recessive OI due to biallelic mutations in collagen I or V genes have usually had severe bone fragility and again no dramatic eye phenotype reported. Recessive *CRTAP* mutations also appear to result in severe bone phenotypes but without significant ophthalmic complications [40], making it likely that these severe recessive OI presentations would be able to be differentiated from BCS.

**Figure 3:**
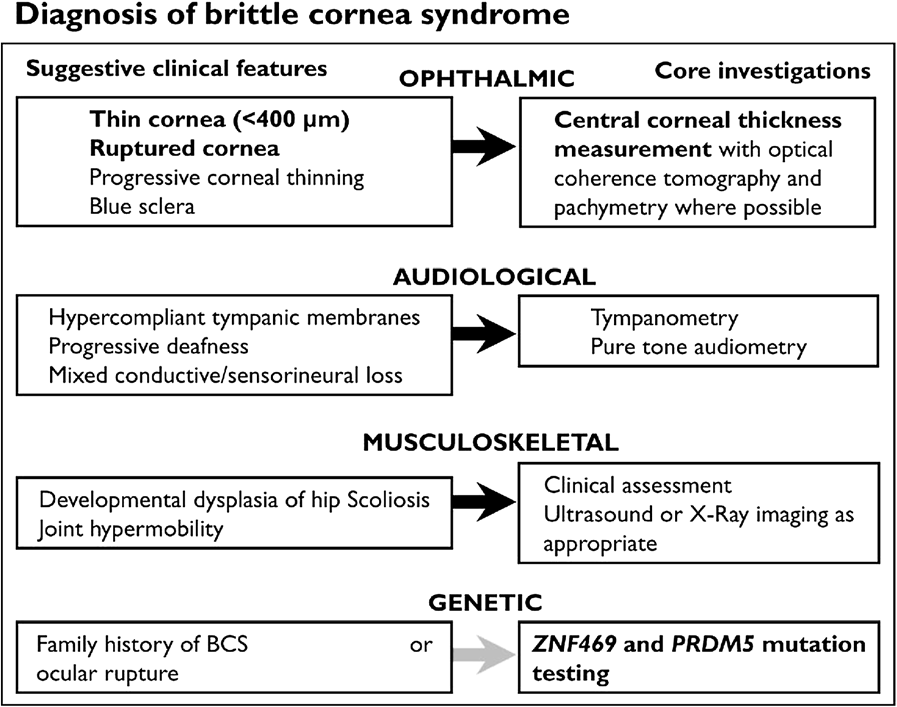
Diagnostic algorithm for patients with suspected brittle cornea syndrome.

Clinical differentiation between EDS VI and BCS may be challenging, but patients with EDS VI frequently have more pronounced generalised connective tissue manifestations. Premature death from arterial or visceral rupture, similar to that seen in EDS type IV (OMIM:130050) is well documented in EDS type VI [15,16], but no such complications have yet been described in BCS. The small numbers of patients identified to date, however, and their predominantly young ages, mean that modestly increased risks for such sequelae cannot currently be excluded. In keeping with more marked generalised connective tissue effects, a greater degree of muscular hypotonia in infancy may be seen in EDS VI [15] than has been recorded in BCS. Similarly, scoliosis may be seen in either condition, but severe early onset scoliosis may be more characteristic of EDS VI [15]. An algorithm to assist diagnosis of BCS is suggested in Figure 3.

Other differential diagnoses for BCS include the musculocontractural form of EDS, another autosomal recessive connective tissue disorder (OMIM: 601776), due to biallelic mutations in *CHST14*[17]. Indeed, one individual whose sample was referred for genetic testing for BCS following corneal rupture was subsequently found to have a homozygous mutation in *CHST14*. Fixed adducted thumbs have been described as a characteristic clinical feature of patients with *CHST14* mutations, in contrast to the 5th finger contractures that result in the mild camptodactyly noted in several patients with BCS (Figure 1b).

Further recently described variant forms of EDS, such as that with progressive scoliosis and deafness (OMIM: 614557) due to biallelic mutations in *FKBP14*, may also show clinical overlap with BCS [18], though the full range of clinical phenotypes due to mutations in these and other newly identified genes requires further definition.

Features that differentiate BCS from EDS type VI are the presence of obvious and dramatic ocular signs in children with BCS, alongside mild or absent features of generalised connective tissue disorder. Overall, ocular sequelae across the different forms of EDS are rare: an early review of 100 individuals with EDS phenotypes did not reveal any serious ophthalmological complications [19]. In a series of 8 cases reported as EDS with ocular manifestations [11], those with serious ocular features were likely to actually have BCS, as demonstrated by the identification of *PRDM5* mutations in two patients of this series [3]. These individuals had corneal fragility and ruptures, blue sclera, keratoconus in early childhood, and also had consanguineous parents, increasing the likelihood of an autosomal recessive disorder [11]. Scleral fragility has been considered a feature of EDS type VI, whereas corneal fragility is a key feature of BCS. Systematic data on CCT measurements in patients with EDS VI are currently lacking, but CCT around or below 400 μm would appear to a potentially robust diagnostic indicator of BCS.

Muscular hypotonia can be pronounced in EDS type VI, leading to significantly delayed motor development. Such severe delay has not been observed in BCS, with either normal or mildly delayed motor milestones being observed. Vascular abnormalities also appear more prevalent in EDS type VI than in BCS.

Other disorders characterised by blue sclera and potential corneal fragility that may occasionally enter the differential diagnosis of BCS include the spondylocheirodysplastic form of EDS (SCD-EDS) (OMIM: 612350) [20,21], osteogenesis imperfecta (OI; OMIM: 166200) and Marfan syndrome (OMIM: 154700) [13]. Blue sclerae were a feature of SCD-EDS in the 3 families reported to date with this condition. However, one of the two probands from another molecularly confirmed affected family developed unilateral lattice corneal dystrophy with keratoconus in her thirties (Dr M Suri, personal communication). Marfan syndrome and most forms of OI show autosomal dominant inheritance, but with a significant proportion of new mutations, so family history may be uninformative, but clinical distinction between these disorders and BCS should nonetheless normally be possible. Both OI and Marfan syndrome have cardinal clinical features which are not characteristically seen in BCS: in OI, a history of recurrent and sometimes spontaneous fractures, and in Marfan syndrome, tendency to aortic dissection, tall stature and ectopia lentis (lens subluxation).

### Gene function, mutational spectrum and correlation with phenotype

**Figure 4:**
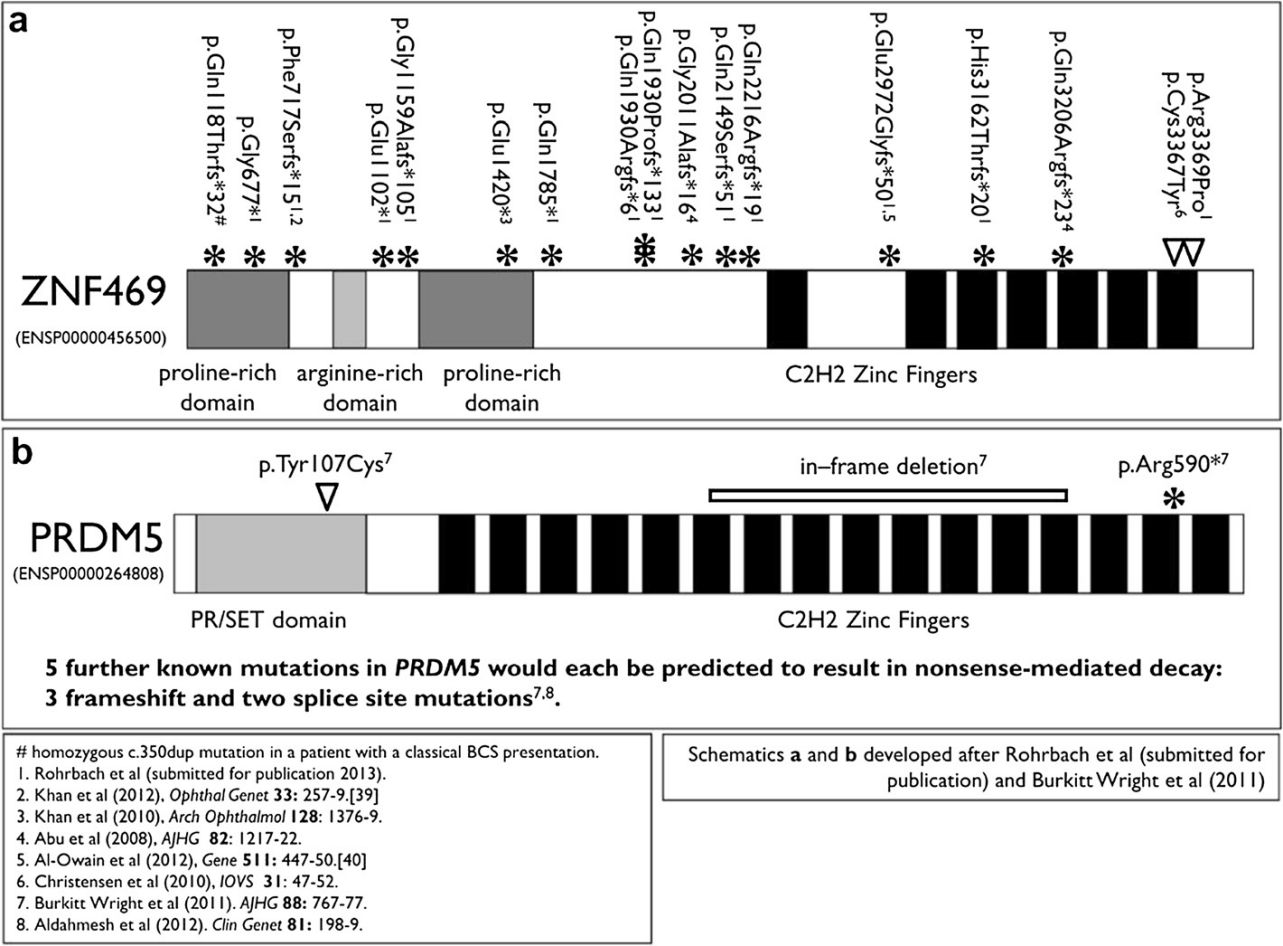
**Mutational spectrum in patients diagnosed with BCS.** Note a greater proportion of mutations in (**a**) *ZNF469* than (**b**) *PRDM5*, and a wide spectrum of mutations consistent with loss-of-function alleles. Articles in which the mutations are described are listed below [23,24].

As can be seen from Figure 4, there is a wide mutational spectrum observed across the two genes known to be responsible for BCS. Both genes encode proteins with multiple zinc fingers, suggesting roles in transcription, and PRDM5 has been identified as a sequence-specific transcription factor [22].

*ZNF469* is a 13 kb open reading frame at 16q24 consisting of a single exon [6] predicting production of a 413 kDa protein of 3953 amino acid residues. Whilst functional data for *ZNF469* is limited, its locus has been repeatedly identified as a top hit in genome-wide association studies for central corneal thickness, confirming its importance in anterior segment development, [25,26] with concomitant implications for pathogenesis of common ocular disorders such as glaucoma [27].

*PRDM5* is a 16 exon gene at 4q25-q26 encoding a 73 kDa protein of 630 amino acid residues. Its role in extracellular matrix (ECM) development has recently been investigated in vivo [28]. PRDM5 was identified to bind to the exonic DNA of collagen I genes and to upstream enhancer elements of proteoglycans with key roles in the ECM, such as decorin, in murine bone [28].

Mutations identified to date in patients with BCS are shown in Figure 4. The high proportion of frameshift and premature truncation mutations across both genes is in itself highly suggestive that these act as loss-of-function alleles. Their functional effects upon components of extracellular matrix have been characterised in patient-derived fibroblasts [3,6]. Total numbers of known affected patients are quite small, but notwithstanding this, there is no current evidence of genotype-phenotype correlation: mutations in either gene appear to cause indistinguishable clinical phenotypes, as shown in Table 2. There is currently no indication that milder or more severe presentations might result from missense substitutions, or other genres of mutation. Similarly, functional assessment of mutated PRDM5 and ZNF469 proteins in skin-derived fibroblasts [3] showed very similar results for each at the RNA level by expression microarray and at the protein level by immunofluorescence. Specific molecular or cellular signatures for phenotypic features, such as high risk of corneal rupture (as in family BCS-001) versus no corneal rupture (family BCS-002), or multiple fractures (BCS-002) versus no fractures (BCS-002), have also not yet been identified [3]. Further evidence regarding the full phenotypic spectrum and genotype-phenotype correlation will emerge as increasing numbers of patients receive a molecular diagnosis, and known patients can be reviewed at more advanced ages. The data available for many patients in the literature is limited, and later onset phenotypes such as scoliosis are likely to be under-represented. Similarly, the presence or absence of later onset features such as retinal detachment or glaucoma in severely compromised eyes may or may not have been recorded for some patients, again leading to underestimates of the incidence of such sequelae in BCS.

**Table 2:** **Common clinical features of BCS in patients with biallelic *ZNF469 *and *PRDM5 *mutations**

**Feature**	**Observed in how many patients (and families) with *ZNF469 *mutations?**	**Observed in how many patients (and families) with *PRDM5 *mutations?**
Ocular rupture	16/19 (8 of 11 families)	9/16 (5 of 8 families)
CCT <400 μm	12/12 (7 families)	9/9 (4 families)
Keratoconus/keratoglobus*	8/12 (7 families)	
Blue sclera	19/19 (11 families)	16/16 (8 families)
Deafness	7/17 (6 of 11 families)	9/16 (3 of 8 families)
Developmental dysplasia of the hip	5/14 (4 of 10 families)	4/16 (3 of 8 families)
Scoliosis	3/18 (2 of 7 families)	3/16 (3 of 8 families)
Small joint hypermobility	12/19 (8 of 11 families)	14/16 (7 of 8 families)

Other features reported in small numbers of affected individuals or families include recurrent fractures, dental abnormalities, learning disability, hypertelorism and orofacial clefting. Such features could represent less common features of BCS or coincidental phenotypes (particularly in individuals from multiply consanguineous pedigrees).

* Whilst keratoconus or keratoglobus have not been noted in all affected individuals, this most frequently appears to be due to extremely early corneal rupture.

### Clinical management

There are many facets to the management of BCS, which are summarised and discussed below. Given the high risk of poor visual outcomes following corneal rupture, recognition of this disorder prior to the occurrence of rupture is a critical step in preserving function and quality of life for affected patients.

Management checklist for patients with brittle cornea syndrome.

OPHTHALMIC: ensure ongoing ophthalmology follow-up

Education and lifestyle advice: patient, family, school and other carers

Protectivepolycarbonate spectacles (activities for which mandated may depend upon history and degree of corneal thinning)

Serial corneal scanning

AUDIOLOGICAL: ensure ongoing audiological follow-up

Serial pure tone audiometry and tympanography

MUSCULOSKELETAL

Newborns and children under 2 years: screening for hip dysplasia

Be alert to other connective tissue phenotypes: monitor for scoliosis

Joint protection advice

OTHER CLINICAL MANAGEMENT

Consider echocardiography, low threshold for cardiac investigation

Be aware of potential pregnancy-related complications

FAMILY IMPLICATIONS

Molecular testing for confirmation of diagnosis

Assessment and genetic testing of other at-risk individuals in family

Consider above interventions for heterozygous mutation carriers

#### Early diagnosis is crucial

Definitive clinical diagnosis of BCS can be difficult, and hence genetic testing for BCS is relevant for affected, possibly affected or at risk individuals, and potential carriers of heterozygous mutations. The condition may be more likely to remain undiagnosed in patients without factors suggestive of autosomal recessive inheritance, for example simplex cases who are children of unrelated parents. It is also possible that penetrating corneal injuries due to BCS, particularly those without a clear history of significant trauma, could be misattributed to non-accidental injury (NAI). Clearly the possibility of NAI must be considered in any such case, but we are aware of allegations of NAI in at least two families affected with BCS (personal communications), in which a child presented initially with corneal rupture. Awareness of the disorder and careful clinical evaluation are required to differentiate NAI from BCS.

#### Prevention of ocular rupture

The key challenge in the management of BCS is the prevention of ocular rupture, which relies on early diagnosis to allow for targeted measures aimed at preventing ocular trauma. A multidisciplinary approach is needed, with provision of both protective polycarbonate spectacles and appropriate education about these and other lifestyle measures for affected individuals, their parents, other caregivers and school staff. As early as 1990, early diagnosis (aged 2 years) of BCS was reported in a patient in whom corneal perforation was averted by the use of special protective glasses [29]. For BCS patients who have very thin corneas, or who have an affected relative who has suffered a corneal rupture, continuous wearing of such eyewear should be recommended, given the multiple identified instances of perforating ocular trauma following the most minor of impacts. For patients with any degree of reduced central corneal thickness, the wearing of protective eyewear should be recommended for active pursuits, but whether continuous use is necessary for these patients is less clear.

#### Optimising visual function

Visual acuity is affected from an early age by keratoconus, keratoglobus and high myopia. Correction of the irregular astigmatism caused by keratoconus and keratoglobus is of limited efficacy. In addition, the use of contact lenses is frequently precluded by extreme corneal thinning [30]. Maximising visual potential in BCS is particularly important given the high proportion of patients with combined visual and hearing loss.

Clinical surveillance of BCS patients for progression of the ocular phenotype is warranted. Progressive corneal ectasia may occur prior to ocular rupture [11] and serial corneal topography can detect progressive thinning. Any evidence of progression would be a strong indication for the provision of protective spectacles, if not already in use. In at least one patient with BCS, a scleral contact lens worn beneath protective spectacles has been considered for refractive correction of keratoconus (personal communication), though the risk-benefit analysis for any such intervention would be different for each individual. In advanced cases of corneal thinning, epikeratoplasty has been advocated in anticipation of corneal rupture. This is a partial thickness corneal graft that aims to increase limbus-to-limbus thickness and permit a full thickness corneal transplant to be performed over it subsequently [30]. This technique is aimed to accommodate the fragility of the recipient bed and disparity between donor and recipient tissue thickness [30,31], preserving globe integrity and improving vision. Javadi et al. [30] performed epikeratoplasty in 7 eyes of 6 patients with advanced keratoglobus or BCS, with mean preoperative corneal thickness of 277 μm. Visual improvements were noted, but so too were significant complications. A neurotrophic ulcer developed in one patient, and epithelial down-growth into the corneal stroma in another patient. Penetrating keratoplasty a few months after epikeratoplasty was therefore advocated for cases of extreme corneal thinning [30,32]. Additional surgical options for extreme corneal thinning include corneo-scleral grafting, which will not improve vision, but may strengthen the peripheral cornea [33].

Collagen crosslinking has been reported to be effective in treating progressive keratoconus in children and adults [34,35]. This technique involves application of riboflavin (vitamin B2) and long wavelength ultraviolet A light (370 nm) to induce chemical reactions in the corneal stroma, resulting in the formation of covalent bonds between the collagen molecules, fibres and microfibrils, strengthening the cornea [36]. Modified collagen crosslinking was recently performed for corneal stabilisation in a child with BCS seen in our clinic, with encouraging preliminary results. Vision improved from 0.05 to 0.16p in one week, and no complications were apparent one month after the procedure. A child with severe corneal manifestations of arterial tortuosity syndrome (ATS) has also been successfully treated in a similar manner: one year after the procedure, reduced corneal curvature and a sustained improvement in visual acuity were observed. CCT in both children was 270 μm.

#### Treatment of corneal rupture

The management of corneal rupture in BCS is challenging. When primary repair has been attempted, this has often been complicated by extensive scarring. When primary repair is not possible, or fails, affected individuals usually require an evisceration. Corneal transplantation to treat extensive scarring after previous rupture has been reported in BCS, but specific complications and considerations have been identified [12]. The efficacy of corneal transplantation in the setting of a diffusely thinned recipient bed is limited. Izquierdo et al. [12] carried out corneal transplantation on a child with BCS and spontaneous corneal rupture. They used a traditional limbus-to-limbus technique but placed sutures further across the recipient cornea in order to prevent “cheese-wiring” through the tissue. Despite this precaution, an intraoperative corneal rupture occurred, when rotation of the sutures to bury the knot from the corneal surface was attempted [12]. A scleral allograft was used to seal the rupture and the child was able to be discharged with a secure wound and visual acuity of 20/100 two weeks postoperatively [12]. However, such limbus-to-limbus corneal grafts are associated with an increased risk of rejection [30]. Macsai et al. [37] described successful emergency epikeratoplasty in a patient with a ruptured cornea and diagnosis of “ocular Ehlers-Danlos syndrome” with normal lysyl hydroxylase levels. No genetic testing has been reported in this patient, but the clinical and biochemical presentation was strongly suggestive of BCS. This was reported as the first successful such procedure, citing the report of Judisch et al. [38] as the first attempt, which ended in enucleation. Similarly, in an early case series of 5 patients with keratoglobus and blue sclera, 2 patients underwent penetrating keratoplasty that was unsuccessful, necessitating subsequent evisceration [31].

#### Management of extraocular manifestations

BCS is a multisystem connective tissue disorder in the great majority of cases, but extraocular manifestations are commonly milder than those seen in many other connective tissue disorders. Data are not available for older patients affected with BCS, but there is currently no evidence for a reduction in life expectancy, in contrast to the high early mortality observed in EDS VI [15] and in many other autosomal recessive connective tissue disorders.

Significant musculoskeletal complications have nonetheless been seen in a high proportion of patients with BCS, particularly developmental dysplasia of the hip (DDH), which has been of unusually late onset in at least one individual (V:4, BCS-002, Burkitt Wright et al.), when it presented in the second year of life following normal neonatal and 6 week checks. This suggests that a high index of suspicion is required in these patients, and that the duration of surveillance for hip dysplasia should perhaps be extended beyond that routinely offered for at-risk infants. Scoliosis is also a common feature of BCS, and therefore affected individuals should be clinically monitored for this, with a low threshold for proceeding to investigations. At present, there are no data to suggest differences in how these and the other musculoskeletal features of BCS should be managed as compared to such complications in the context of other generalised connective tissue disorders.

Heterozygous mutation (carrier status) in one of the genes mutated in BCS may be associated with mild ocular and musculoskeletal manifestations. Both keratoconus and high myopia have been identified in known heterozygous carriers and comprehensive ocular examination is indicated in individuals with (or at risk for) such mutations.

Whilst there is currently little evidence on the effects of being a heterozygous mutation carrier, these genotypes may also confer an additional risk of significant musculoskeletal manifestations. Any potential increased risk of DDH warrants particular attention, and a screening schedule similar to that recommended for babies with a family history of DDH may be appropriate. Whilst specific evidence to support this is currently not available, significant hypermobility has been observed in several heterozygous individuals, suggesting that their individual risks of developing DDH may indeed be elevated compared to the general population.

## Conclusions

We consider it likely that BCS remains an under-recognised condition. In support of this assertion, only one patient with BCS due to compound heterozygous mutations has been identified to date (Patient P2 of Rohrbach et al. [6]). BCS is a condition that is important to recognise, in order to permit appropriate management, including avoidance of misattribution of corneal damage to non-accidental injury, and to facilitate genetic counselling. With the identification of two genes underlying BCS, molecular testing is now available and effective, with a mutation demonstrable in over 95% of cases with a classical presentation. Definitive diagnosis allows for appropriate anticipatory management, including advice and aids to prevent future ocular rupture and careful assessment for combined sensory loss, with its major implications for quality of life.

## Competing interests

The authors declare that they have no competing interests.

## Authors’ contribution

EMMBW, LFP, FDCM and GCMB wrote the body of the paper, but all authors have contributed to the writing of this article. JCS, LA, FLM, SS, MS and MR provided specific clinical data on their patients, including operative details, clinical photographs and investigation results, and opinions about management and differential diagnosis. HLS performed sequencing of BCS-associated genes to identify mutations described in the report. All authors read and approved the final manuscript.
